# PET/CT-guided versus CT-guided percutaneous core biopsies in the diagnosis of bone tumors and tumor-like lesions: which is the better choice?

**DOI:** 10.1186/s40644-019-0253-1

**Published:** 2019-10-29

**Authors:** Min-hao Wu, Ling-fei Xiao, Huo-wen Liu, Zhi-qiang Yang, Xiao-xiao Liang, Yan Chen, Jun Lei, Zhou-ming Deng

**Affiliations:** Department of Spine Surgery and Musculoskeletal Tumor, Zhongnan Hospital of Wuhan University, 168 Donghu Street, Wuchang District, Wuhan City, 430071 Hubei Province People’s Republic of China

**Keywords:** Image-guided bone biopsy, FDG PET/CT, CT-guided, Bone lesions, Diagnostic performance, Complications

## Abstract

**Objective:**

The present study aimed to evaluate the diagnostic performance and safety of PET/CT-guided percutaneous core bone biopsy and to compare the PET/CT-guided method to conventional CT-guided percutaneous core biopsies to diagnose Chinese patients with bone tumors and tumor-like lesions.

**Methods:**

Data for 97 patients with bone tumors and tumor-like lesions diagnosed by percutaneous core bone biopsy from February 2013 to November 2018 were retrospectively analyzed. The study included 42 cases in the PET/CT group and 55 cases in the CT alone group. The diagnostic performance, cost and complications associated with the intervention were compared between the two groups. All patients were eventually confirmed to have bone tumors and tumor-like lesions according to surgical pathology findings.

**Results:**

There were no significant differences in patient characteristics (*P* > 0.05). For the patients in the PET/CT group, the overall diagnostic yield of the initial biopsies and the diagnostic accuracy derived from the surgically proven cases were both 97.62%, which was significantly higher than the values in the CT group during the same period (*P* < 0.05). No major biopsy-related complications (e.g., serious bleeding or tumor dissemination) occurred before, during, or after the intervention. Therefore, no significant difference was observed between the two groups with regard to the complication rate (*P* > 0.05).

**Conclusion:**

Compared with CT-guided percutaneous bone biopsy, PET/CT-guided percutaneous bone biopsy is an effective and safe alternative with high diagnostic performance in the evaluation of hypermetabolic bone lesions to diagnose bone tumors and tumor-like lesions.

## Introduction

Currently, traditional computed tomography (CT)-guided percutaneous core-needle biopsy is the gold standard diagnostic procedure for patients with newly developed bone lesion s[1]. Numerous publications have reported that CT-guided percutaneous core-needle biopsy is a well-established technique and is effective for the initial diagnosis of musculoskeletal lesions, with a measured diagnostic yield ranging between 70 and 89% and a reported accuracy between 61 and 98% [2–11]. These results confirm the high sensitivity of CT-guided percutaneous needle biopsy for the diagnosis of bone tumors through the use of a minimally invasive procedure, which is associated with cost savings and fewer biopsy complications than open surgical biopsy [6, 7, 10–14]. However, the disadvantage of this guided biopsy method is that metabolically active lesions without distinctive morphology may not be reliably assessable by CT-guided biopsy, and the false-negative biopsy rate of such lesions may be substantially higher with CT-guided biopsy [15]. Recent studies have demonstrated the effective diagnostic yield of PET/CT-guided percutaneous needle biopsy for bone tumors, with a first-time diagnostic success rate as high as 96.1% and an ideal 100% overall diagnostic success rate and sensitivity [16–20]. PET/CT combines the anatomic information from CT with the metabolic characterization from PET; the use of PET guidance improves target selection and allows a direct biopsy needle trajectory to the hypermetabolic region of active isolated or multifocal (≥2) bone lesions, which is more likely to yield a positive biopsy result [16].

Whether PET/CT-guided biopsy can replace routine CT-guided biopsy to diagnose bone neoplasms remains controversial [21]. The purpose of this study was to compare the diagnostic yield, accuracy, cost and safety of PET/CT-guided and CT-guided percutaneous core biopsies in diagnosing bone tumors and tumor-like lesions during the same period.

## Materials and methods

### Patients and groups

We conducted a retrospective study of 97 patients who had undergone image-guided biopsy at our center from February 2013 to November 2018. The inclusion criteria were as follows: (1) treatment by open surgery with tumor resection after biopsy; (2) primary percutaneous core-needle biopsy was performed at our center; (3) ^18^F-FDG PET/CT-guided or CT-guided biopsy was performed as part of the initial diagnostic intervention before open surgery; and (4) final surgical histopathology results were confirmed as bone tumors or tumor-like lesions. According to the electronic medical records and pathological reporting systems, we identified the clinical characteristics, tumor size, tumor location, classification, and biopsy modalities recorded during and after the procedure. To determine whether PET/CT-guided or CT-guided biopsy should be performed for each patient, we typically conducted a multidisciplinary team meeting (MDT) with our experienced histopathologists, radiologists and oncologic orthopedic surgeons to assess imaging presentation, including X-ray, CT, magnetic resonance imaging (MRI), or bone scintigraphy, before any intervention. In detail, for patients with suspected bone metastases or primary bone malignancies, PET/CT-guided biopsy may be initially preferred.

### Image-guided percutaneous core-needle biopsy procedures

**Fig. 1:**
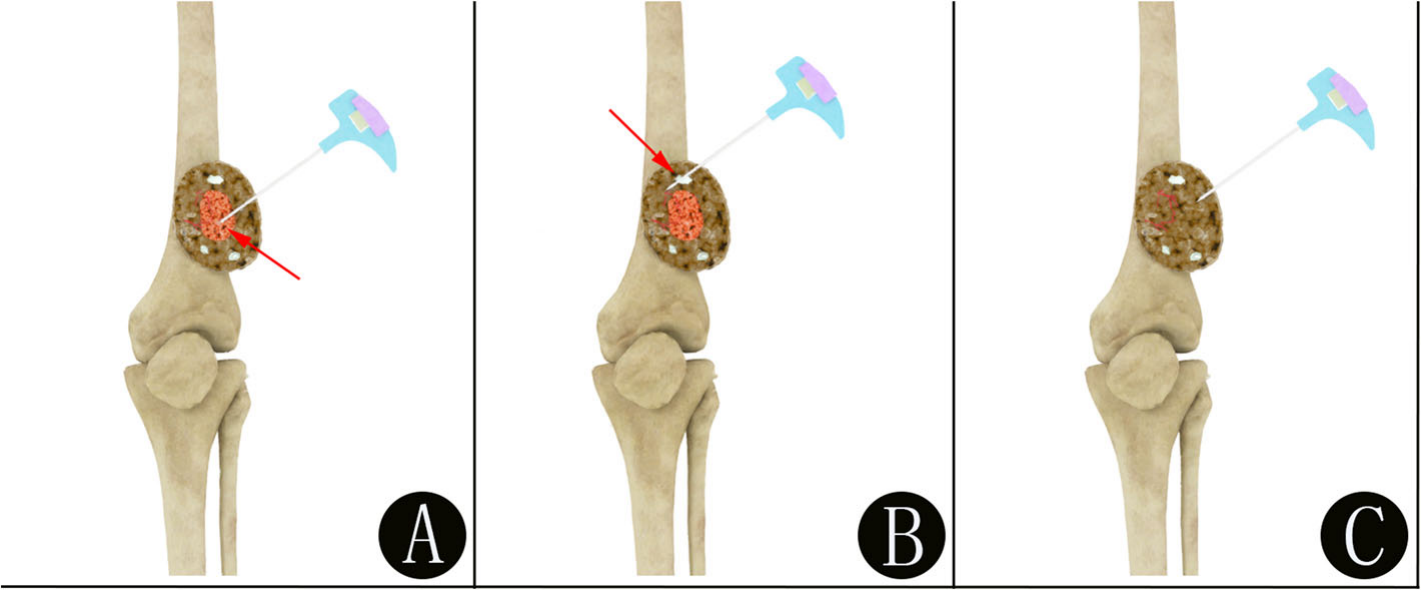
Schematic diagram of the two methods for performing imaging-guided bone biopsy (**a-c**). **a** The demonstration of a precise PET/CT-guided biopsy for bone lesions by guiding the needle placement in the metabolically active portion (red arrow) of the bone lesion. **b** The demonstration of a false PET/CT-guided biopsy for bone lesions by guiding the needle placement in the reactive areas (red arrow) with necrosis or fibrosis, which may lead to a false-negative biopsy. **c** The demonstration of a CT-guided biopsy for bone lesions

All patients were informed of the procedure and its potential complications before undergoing biopsy. In our center, the biopsy procedure is generally conducted under local anesthesia alone (lidocaine 1.0% without epinephrine), but for patients who experience nervousness, anxiousness, uncooperativeness or intolerance to pain, a conscious sedation/analgesia protocol might be required. Additionally, a senior anesthesiologist electively checked the patient and attended the procedure to monitor the patient’s vital signs throughout the procedure. A schematic diagram of the two methods for bone biopsy of a bone lesion is shown in Fig. 1. All of these procedures were performed by the same team with least 3 years of experience in interventional bone oncology.

#### ^18^F-FDG PET/CT-guided biopsy group

First, whole-body FDG PET/CT imaging was performed following intravenous injection of 222–370 MBq (6–10 mCi) ^18^F-FDG for all patients. The emission images were acquired within 60–90 min of tracer injection using an integrated PET/CT system (Discovery VCT64; GE Medical Systems, Milwaukee, Wisconsin, USA). Areas of nonphysiologically enhanced ^18^F-FDG uptake over the background were classified as positive for bone lesions. Patients were positioned in a prone or supine position before receiving either local anesthesia alone or a combination of conscious sedation/analgesia. The access path was defined based on the PET/CT presentation of the metabolically active bone lesion and its relationship to anatomic structures. Interventions were performed under strictly aseptic conditions by our bone oncologists using a step-by-step technique according to a previously published protocol [21, 22].

**Fig. 2:**
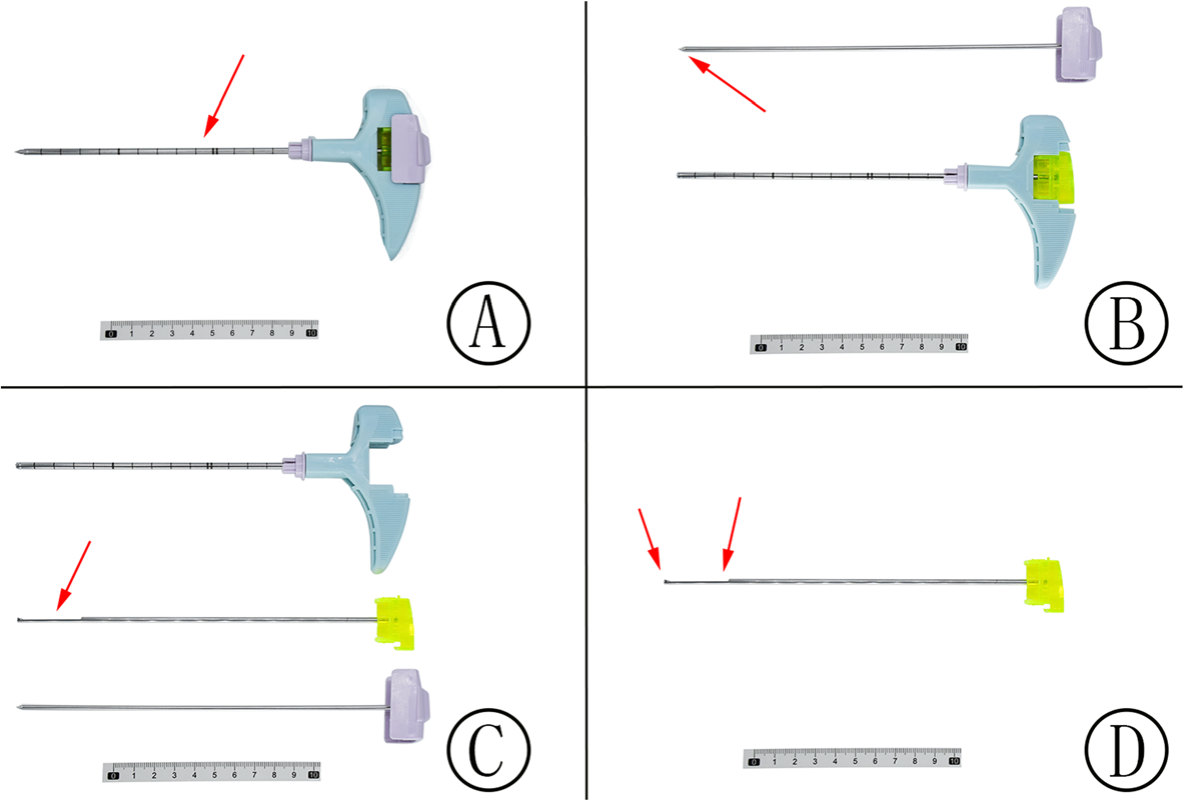
Paragon bone biopsy systems (**a-c**). The system consists of a 9-gauge external cannula (**a**, red arrow) with an internal drill (**b**, red arrow) and a 12.5-gauge trephine biopsy needle (**c-d**, red arrow). When the external cannula and drill were inserted into the bone lesion, the internal drill was replaced by the trephine biopsy needle (**c**, red arrow). Core biopsies were then extracted through the trephine biopsy needle (**d**, red arrow)

**Fig. 3:**
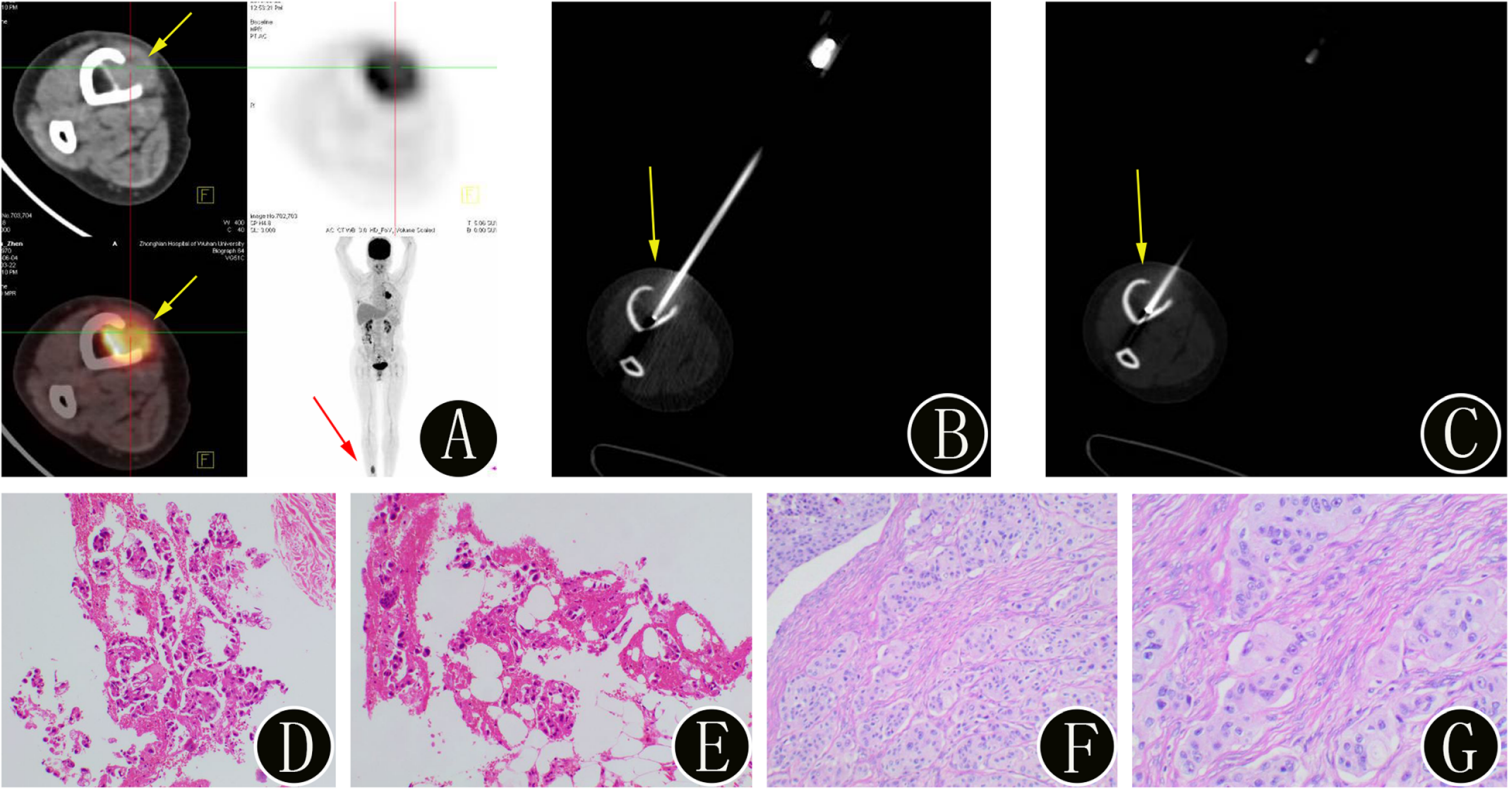
A 45-year-old woman with suspected lung carcinoma and newly identified bone lesion. **a** ^18^F-FDG PET/CT imaging shows uptake (SUVmax 6.4) in the bone lesion in the right tibia (yellow arrow). The maximum intensity projection image also confirms the presence of the bone lesion (red arrow). **b-c** The intraprocedural axial noncontrast CT image (using bone windows) shows the biopsy needle targeted within the lesion (yellow arrow). The histopathologic biopsy results (**d**: hematoxylin and eosin, original magnification 40×, **e**: hematoxylin and eosin, original magnification 100×) confirmed the bone lesion as a metastatic adenocarcinoma. Immunohistochemistry showed cancer cells: TTF-1 (+), PAX8 (−), and CK7 (+). Finally, the surgical histopathology results (**f**: hematoxylin and eosin, original magnification 40×, **g**: hematoxylin and eosin, original magnification 100×) of the bone lesion confirmed the diagnosis of bone metastases

**Fig. 4:**
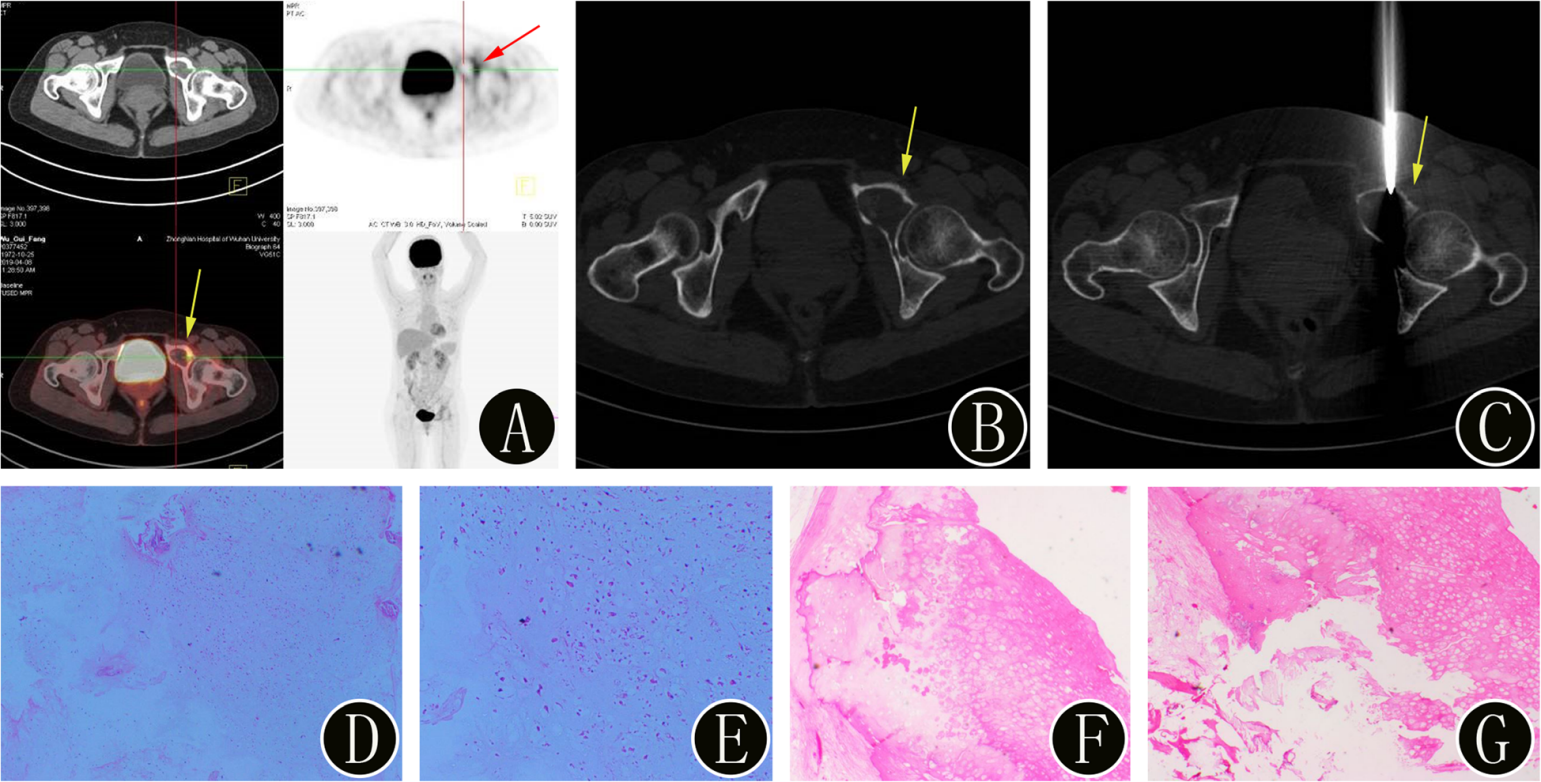
A 46-year-old woman with suspected primary bone malignancies of the pelvis. **a** ^18^F-FDG PET/CT imaging shows uptake (SUVmax 5.1) in a left pubic lesion (yellow arrow). The axial PET image also confirms the presence of the bone lesion (red arrow). **b-c** The intraprocedural axial noncontrast CT image (using bone windows) shows the biopsy needle targeted within the lesion (yellow arrow). The histopathologic biopsy results (**d**: hematoxylin and eosin, original magnification 40×, **e**: hematoxylin and eosin, original magnification 100×) confirmed the bone lesion as a highly differentiated chondrosarcoma. Finally, the surgical histopathology results (**f**: hematoxylin and eosin, original magnification 40×, **g**: hematoxylin and eosin, original magnification 100×) of the bone lesion confirmed the diagnosis of a highly differentiated chondrosarcoma (WHO grade I)

**Fig. 5:**
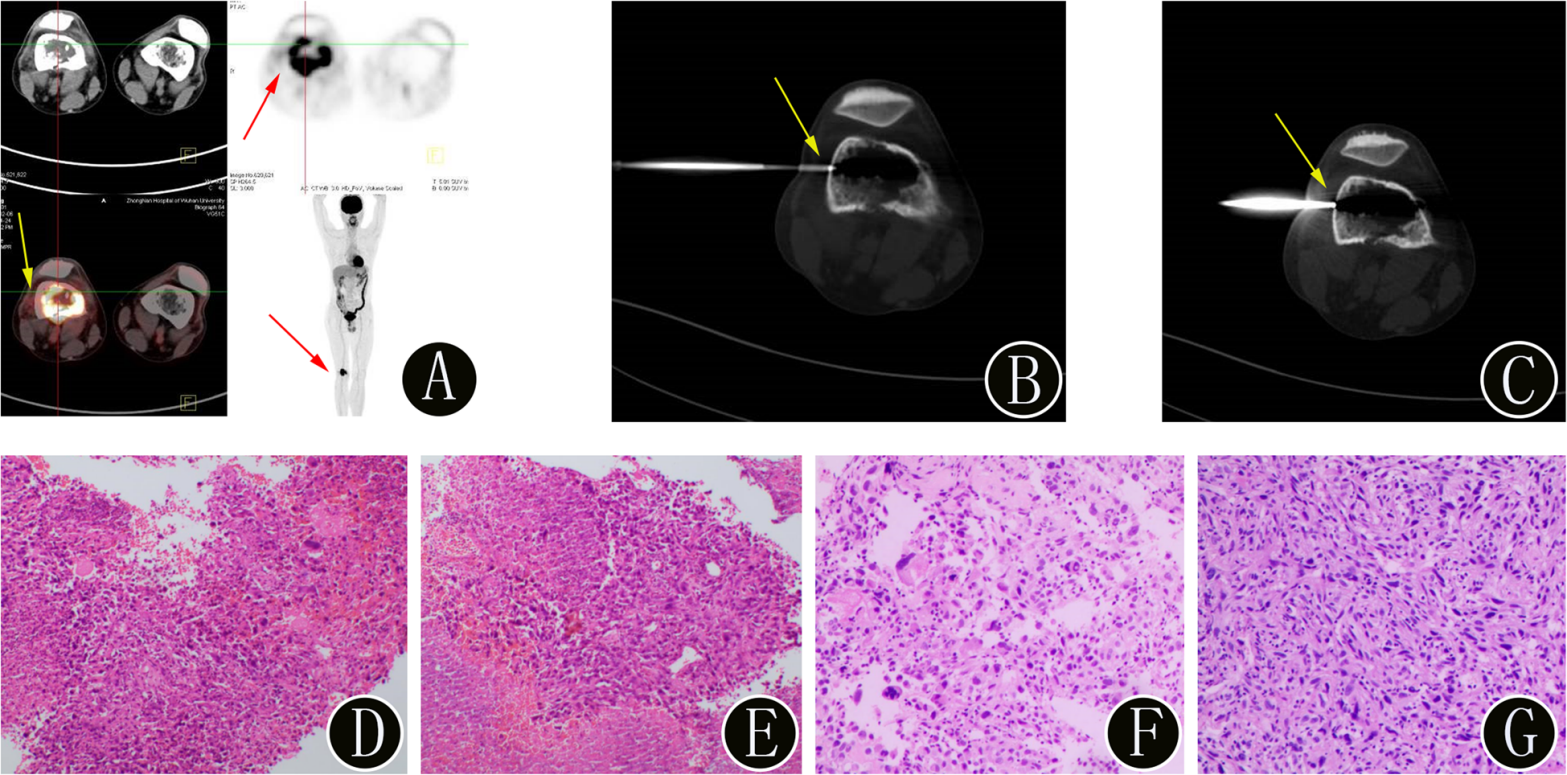
A 45-year-old man with suspected primary bone malignancies of the right femur. **a** ^18^F-FDG PET/CT imaging shows uptake (SUVmax 22.7) in the bone lesion in the right femur (yellow arrow). The maximum intensity projection image also confirms the presence of the bone lesion (red arrow). **b-c** The intraprocedural axial noncontrast CT image (using bone windows) shows the biopsy needle targeted within the lesion (yellow arrow). The histopathologic biopsy results (**d**: hematoxylin and eosin, original magnification 40×, **e**: hematoxylin and eosin, original magnification 100×) confirmed the bone lesion as an osteosarcoma. Immunohistochemistry showed cancer cells: S-100 (−), MDM-2 (−), SATB2 (+), P16 (−), CDK4 (weak +), CK (−), VIMENTIN (+), LCA (−), Desmin (−), MyoD1 (−), and Ki-67 (Li: 50%). Finally, the surgical histopathology results (**f**: hematoxylin and eosin, original magnification 40×, **g**: hematoxylin and eosin, original magnification 100×) of the bone lesion confirmed the diagnosis of osteosarcoma

Under the guidance of the fused PET/CT and CT imaging scan, the biopsy needle was introduced in a stepwise manner. At our center, the biopsy system usually used a Paragon bone biopsy system (Paragon Bone Biopsy Systems, Sterylab, Italy), which consists of a 9-gauge external cannula with an internal drill and a 12.5-gauge trephine biopsy needle (Fig. 2). The external cannula and drill were inserted into the bone lesion, and the internal drill was then replaced by the trephine biopsy needle. The needle angle and direction were adjusted according to the position of the metabolically active bone lesion. For each patient, at least one sample was collected and sent to the laboratory. All patients were then observed for at least 2 h after the biopsy to ensure hemodynamic stability, and their respiratory condition was monitored. Representative ^18^F-FDG PET/CT images of patients who underwent bone biopsy are shown in Figs. 3, 4 and 5.

#### CT-guided biopsy group

**Fig. 6:**
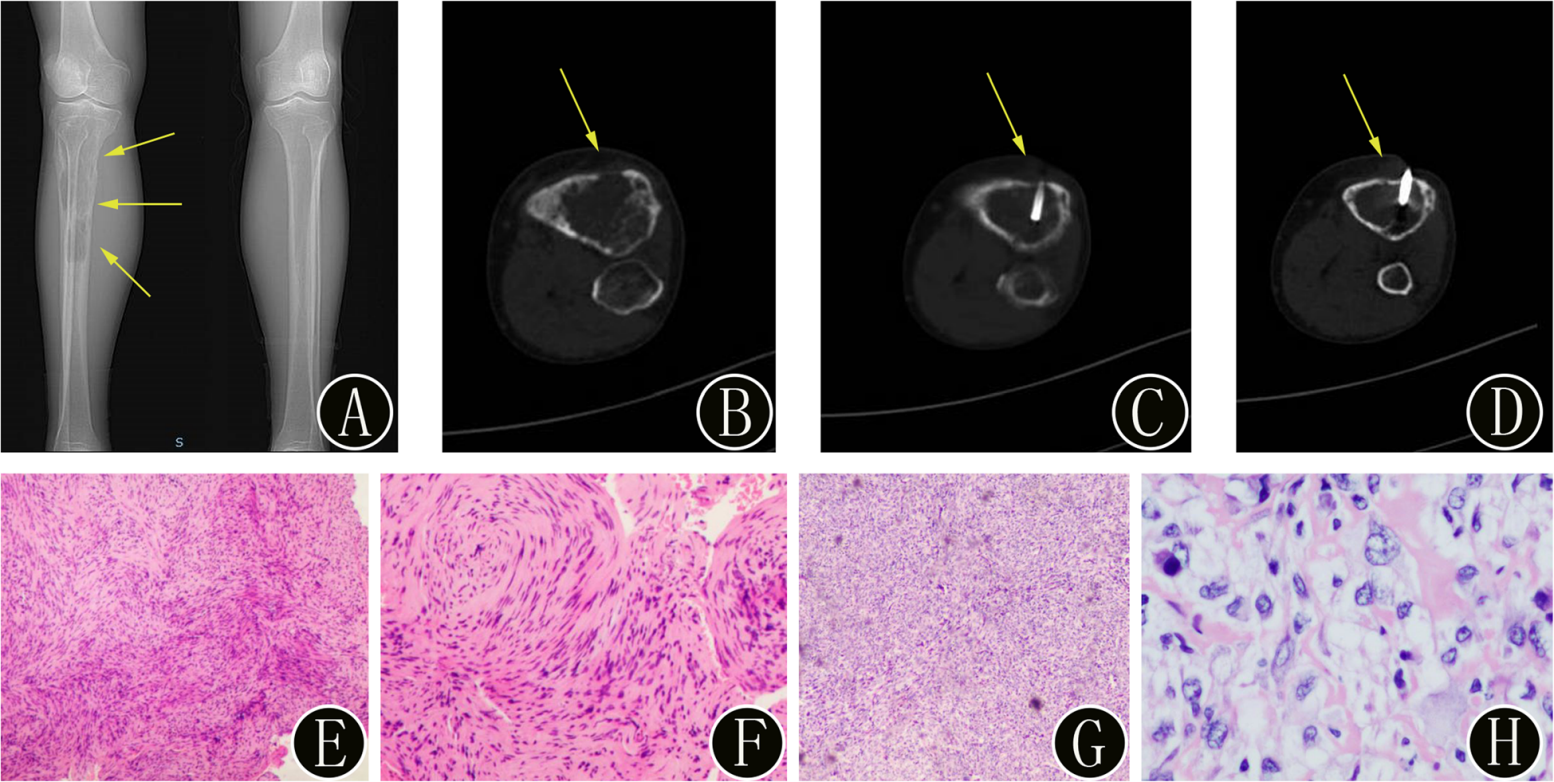
A 56-year-old woman with misdiagnosed primary benign bone tumors of the right tibia. **a** Preoperative anteroposterior plain radiographs demonstrate expansile, osteolytic bone destruction without periosteal reactive new bone (yellow arrow). **b-d** The intraprocedural axial noncontrast CT image (using bone windows) shows that the biopsy needle is inserted into the bone lesion (yellow arrow). The histopathologic biopsy results (**d**: hematoxylin and eosin, original magnification 40×, **e**: hematoxylin and eosin, original magnification 100×) diagnosed the bone lesion as a benign fibrous histiocytoma. Immunohistochemical staining: CD163 (+), CD34 (−), CD68 (KP1) (+), Desmin (−), Ki-67 positive rate approximately 2%, S ≤ 100 (−), SATB2 (−), SMA (+), and STAT6 focus (+). However, the surgical histopathology results (**f**: hematoxylin and eosin, original magnification 40×, **g**: hematoxylin and eosin, original magnification 100×) of the bone lesion confirmed a diagnosis of osteosarcoma. Immunohistochemistry showed that tumor cells were BCL-2 (focus +), Caldesmon (+), Calponin (+), CD34 (−), CD99 (+), Desmin (−), FLI-1 (focus +), HMB45 (−), Ki-67 (Li: 10%), Muc4 (−), S ≤ 100 (−), SMA (+), SOX-10 (−), STAT6 (−), and SATB2 (+)

**Fig. 7:**
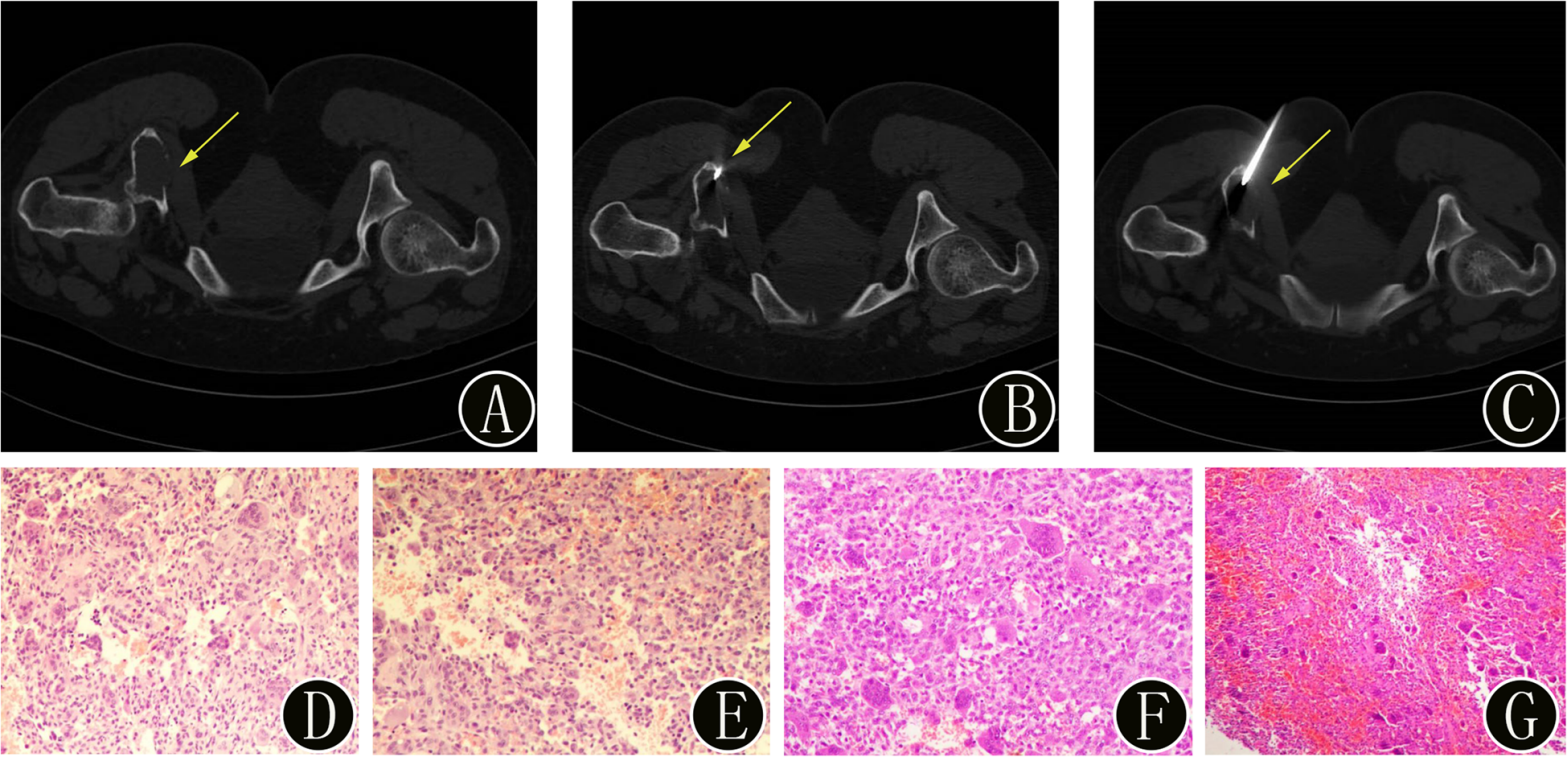
A 42-year-old woman with suspected primary benign bone tumors of the pelvis. **a** Preoperative axial CT image demonstrates osteolytic bone destruction in the fifth lumbar vertebra (yellow arrow) and expansile, osteolytic bone destruction with cortical interruption (yellow arrow). **b-c** The intraprocedural axial CT image (using bone windows) shows that the biopsy needle is inserted into the bone lesion (yellow arrow). The histopathologic biopsy results (**d**: hematoxylin and eosin, original magnification 40×, **e**: hematoxylin and eosin, original magnification 100×) diagnosed the bone lesion as a giant-cell tumor. Finally, the surgical histopathology results (**f**: hematoxylin and eosin, original magnification 40×) of the bone lesion confirmed the diagnosis of a giant-cell tumor. Immunohistochemistry showed that the monocyte-like cells were CD163 (+), CD68 (KP1) (+), Ki-67 (positive rate approximately 40%), p63 (+), and SMA (−)

**Fig. 8:**
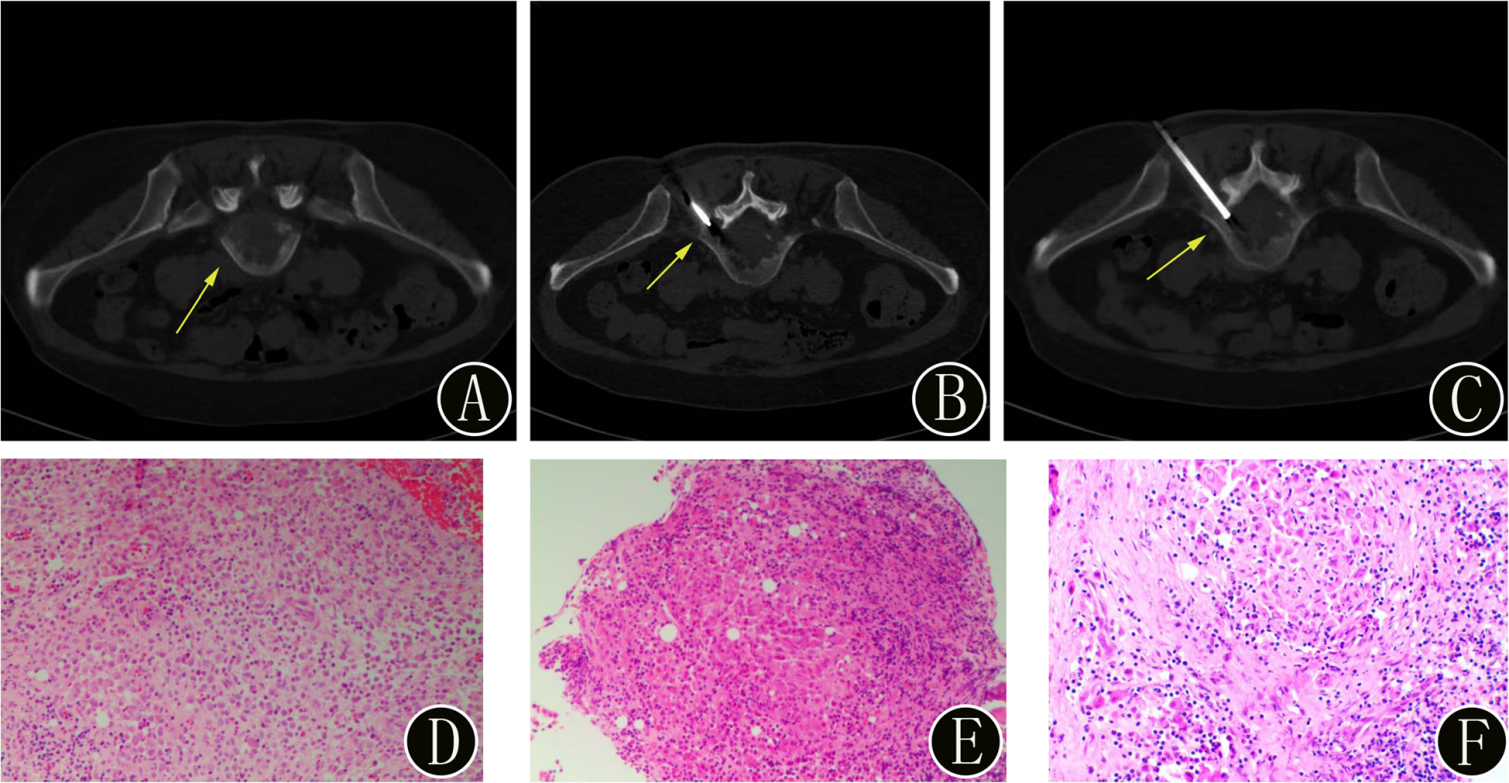
A 56-year-old woman with suspected primary benign bone tumors of the lumbar vertebra. **a** Preoperative axial CT image demonstrates osteolytic bone destruction in the fifth lumbar vertebra (yellow arrow). **b-c**: The intraprocedural axial CT image (using bone windows) shows that the biopsy needle is inserted into the bone lesion (yellow arrow). The histopathologic biopsy results (**d**: hematoxylin and eosin, original magnification 40×, **e**: hematoxylin and eosin, original magnification 100×) diagnosed the bone lesion as Langerhans cell histiocytosis. Immunohistochemistry showed that the monocyte-like cells were S ≤ 100 (+), CD1a (+), Langerin (+), CD68 (KP1) (−), CD163 (+), CK (−), and LCA (+); the positive rate of Ki-67 was approximately 10%, and acid fast staining was negative. Finally, the surgical histopathology results (**f**: hematoxylin and eosin, original magnification 40×) of the bone lesion confirmed the diagnosis of Langerhans cell histiocytosis. Immunohistochemistry showed that the cells were CD1a (+), S ≤ 100 (+), Langerin (+), CD68 (KP1) (weak +), and Ki-67 (Li 20%)

Image guidance was provided by CT (SOMATOM Emotion 16; Siemens Healthcare, Erlangen, Germany). Generally, the patient was positioned on the CT table in a practical way to access the lesion to be biopsied while maximizing comfort for both the patient and operator. Following an initial CT scan of the bone lesion, the biopsy path was determined as per the prior multidisciplinary meeting discussion. After local anesthetic, the biopsy procedures were performed using a standard coaxial technique to allow multiple passes through a single skin puncture and a single track. A coaxial needle was inserted in the previously set position. The needle angle and direction were adjusted according to the position of the suspected lesion. Three or four specimens were collected. The remaining procedures were the same as those for PET/CT-guided biopsy. Representative CT images of patients who underwent bone biopsy are shown in Figs. 6, 7 and 8.

### Evaluation of diagnostic performance and cost

The diagnostic performance was assessed in terms of diagnostic yield and accuracy, which were determined for bone tumors and tumor-like lesions in the two groups by comparing the bone biopsy results with those of the gold standard diagnostic test: histopathological examination after surgical lesion resection. Moreover, the study covered the costs of hospitalization associated with bone biopsy, including the daily costs associated with personnel, drugs, medical devices, operating room use (including consumables and clinical facilities),laboratory tests, maintenance and overhead. The financial department of our center provided the sources of the total costs. All costs were managed by the China Food and Drug administration (CFDA) and are given in RMB.

### Safety evaluation

Safety was evaluated by monitoring the bone biopsy-related complications in patients within 3 weeks after treatment. Local complications associated with the bone biopsy were recorded during the follow-up period and included serious bleeding, severe pain, hematoma, vessel or nerve injury, infection, wound dehiscence and, most seriously, tumor dissemination.

## Statistical analysis

All continuous variables are expressed as the mean ± SD. Independent-sample t-tests and Pearson’s chi-square tests were used for group comparisons of continuous and categorical variables. Statistical significance was set at *P* < 0.05. All statistical analyses were completed using SPSS (version 22.0; IBM, Chicago, IL).

## Results

### Clinical characteristics

During the study period, 97 patients who were diagnosed with bone tumors or tumor-like lesions were eventually enrolled in this retrospective study, including 42 treated by PET/CT-guided biopsy and 55 treated by CT-guided biopsy. Detailed data concerning the patients and the biopsy sites of each biopsy guidance group are shown in Table 1. There were no significant differences in the clinical and demographic characteristics between the two groups.

**Table 1:** Comparison of the baseline clinical characteristics of the patients in the PET/CT and CT groups

Variable	PET/CT group (*n* = 42)	CT group (*n* = 55)	χ^2^ or t value	*P* value
Sex				
Male	30	34	0.98	0.322^*^
Female	12	21		
Age (mean + SD)	57.6 ± 5.2	55.7 ± 7.3	0.275	0.583^#^
Localization of the bone biopsy				
Spinal column				
Cervical vertebrae	2	1	0.653	0.419^*^
Thoracic vertebrae	5	5	0.204	0.652^*^
Lumbar vertebrae	7	10	0.038	0.846^*^
Sacrum	6	7	0.05	0.823^*^
Upper extremity				
Humerus	5	5	0.204	0.652^*^
Radius	2	1	0.653	0.419^*^
Ulna	1	2	0.125	0.723^*^
Lower extremity				
Femur	4	8	0.554	0.457^*^
Tibia	2	6	1.189	0.275^*^
Fibula	2	2	0.076	0.782^*^
Pelvis	6	8	0.001	0.971^*^
Number of bone lesions				
Isolated	17	25	0.24	0.624^*^
Multifocal (≥2)	25	30		
Lesion diameter (mean ± SD cm)	5.5 ± 1. 8	5.1 ± 2.5	2.683	0.753^#^
Postsurgical histopathology diagnosis				
Primary benign bone tumor	3	10	2.5	0.114^*^
Primary bone malignancies	19	18	0.957	0.328^*^
Bone metastases	17	20	0.171	0.679^*^
Tumor-like bone lesion	3	7	0.803	0.370^*^

*χ^2^ test, ^#^Independent-samples t-test

### Diagnostic performance and cost

All bone biopsies were successfully performed, and specimens for histological and immunohistochemical examinations were obtained after the biopsy and surgical procedure. The final pathological findings after surgical resection of the tumor for each biopsy guidance group are shown in Table 2. No significant difference was observed between PET/CT-guided and CT-guided percutaneous biopsies with regard to postsurgical pathological type (Table 2, *P* > 0.05). To directly compare the bone biopsy results and the gold standard examination results, we evaluated the overall diagnostic yield and accuracy between the PET/CT and CT groups for diagnosing bone tumors and tumor-like lesions (Table 3).

**Table 2:** Comparison of the pathological findings after open surgery between the PET/CT and CT groups

Postsurgical histopathology diagnosis	PET/CT group (n = 42)	CT group (n = 55)	χ^2^ value	P value
Primary benign bone tumors				
Giant-cell tumor	2	3	1.311	0.252
Osteoblastoma	1	2	0.231	0.631
Osteoid osteoma	0	2	0.709	0.4
Fibrous histiocytoma	0	3	1.17	0.279
Primary bone malignancies				
Osteosarcoma	4	3	0.116	0.734
Chondrosarcoma	2	5	1.793	0.181
Chordoma	5	2	1.393	0.238
Ewing’s sarcoma	1	1	0.002	0.969
Malignant fibrous histiocytoma	0	2	2.232	0.135
Lymphoma	4	3	0.116	0.734
Myeloma	3	2	0.173	0.677
Bone metastases				
Lung tumor	7	9	0.055	0.815
Breast tumor	2	1	0.564	0.452
Kidney tumor	2	3	0.082	0.774
Prostate tumor	1	2	0.209	0.647
Colorectal tumor	3	3	0.047	0.828
Stomach tumor	2	2	0.03	0.863
Tumor-like lesions				
Simple cyst	0	2	1.559	0.212
Aneurysmal bone cyst	1	3	0.569	0.451
Langerhans cell histiocytosis	2	2	0.076	0.782

**Table 3:** Comparison of the diagnostic performance and cost between the PET/CT and CT groups

	Biopsy method		λ^2^ value	P value
	PET/CT group	CT group		
Overall diagnostic yield	97.62% (41/42)	81.82% (45/55)	4.825	0.028^*^
Diagnostic accuracy	97.62% (41/42)	76.36% (42/55)	6.739	0.009^*^
Average cost (mean + SD, Thousand RMB)	13 ± 5.5	4.3 ± 3.1	2.352	< 0.001^#^

^*^χ^2^ test, ^#^Independent-samples t-test

Of the 42 lesions biopsied under PET/CT guidance, we deemed one core-needle biopsy unsatisfactory due to the absence of tumor cells (bone metastases originating from the prostate). Total concordance between the bone biopsy results and the surgical pathology findings was found in the remaining 41 lesions. The surgical histopathology results showed primary benign bone tumors in 3 cases (7.14%), primary bone malignancies in 19 (45.24%), bone metastases in 17 (40.48%), and tumor-like lesions in 3 (7.14%). The average cost for each patient in the PET/CT group was 13 ± 5.5 thousand RMB. Of the 55 CT-guided biopsies, we deemed ten core-needle biopsies unsatisfactory due to the absence of tumor cells (two chondrosarcomas, one osteosarcoma, one Langerhans cell histiocytosis, two lymphomas and two bone metastases originating from the colorectum and lung) and excess necrotic tissue (one malignant fibrous histiocytoma and one fibrous histiocytoma). Total concordance between the bone biopsy results and the surgical pathology findings was found in the remaining 42 lesions. The other 3 cases of discordance between the initial biopsy results and surgical procedure findings were one osteosarcoma (bone biopsy: fibrous histiocytoma, Fig. 6) and two giant-cell tumors (bone biopsies: aneurysmal bone cyst and chondroblastoma). The surgical histopathology results showed primary benign bone tumors in 10 cases (18.18%), primary bone malignancies in 18 (32.73%), bone metastases in 20 (36.36%), and tumor-like lesions in 7 (12.73%). The average cost for each patient in the CT group was 4.3 ± 3.1 thousand RMB.

Both the overall diagnostic yield and the accuracy for PET/CT-guided bone biopsy were 97.62% (41/42), and the overall diagnostic yield and accuracy for CT-guided bone biopsy were 81.82% (45/55) and 76.36% (42/55), respectively. A significant difference was observed between the PET/CT and CT groups with regard to diagnostic performance (*P* < 0.05). Moreover, in our center, a significant difference was noted in the average cost of bone biopsy between the two groups during the same period (*P* < 0.001) (Table 3).

### Safety

No major biopsy-related complications (e.g., serious bleeding or tumor dissemination) occurred before, during, or after the intervention, but slight bleeding and pain were encountered in almost all patients who underwent biopsy. Complications such as hematoma were limited to two patients who underwent CT-guided bone biopsies. No other complications and no deaths occurred among our current study population. Therefore, there was no significant difference in complication rate between the PET/CT and CT groups (*P* > 0.05).

## Discussion

The main finding of our study is that PET/CT-guided biopsy was better than CT-guided percutaneous bone biopsy for the diagnosis of bone tumors and tumor-like lesions, with significant differences between the two groups in regard to diagnostic performance. Safety, namely, the incidence of complications following intervention, did not differ between the two treatment groups. There were no major complications in this study. Percutaneous core biopsy using imaging guidance is a well-established technique in routine clinical settings [10]. Over the past few decades, CT-guided percutaneous biopsy was often recommended in the initial diagnosis of skeletal lesions; this procedure has been accepted as safe, minimally invasive and cost-effective for providing diagnostic confirmation, thereby preventing the need for more risky and invasive open surgical biopsy procedures in most patients [5, 6, 10]. Previous studies have also described the success rate of CT-guided biopsy in identifying morphologically clear bone lesions to be in the range of 69–90%, but the success rate of biopsies may be unexpectedly lower for lesions that are characterized by their metabolic information rather than by anatomic structures (Fig. 6 )[1, 18, 19, 22]. Furthermore, the diagnostic performance, cost and safety of CT-guided biopsies for bone tumors and tumor-like lesions remain controversial when compared with those of PET/CT-guided biopsies. In contrast to a traditional CT-guided biopsy, an ^18^F-FDG PET/CT-guided biopsy can target the metabolically active area or portion of a bone lesion [16]. In a prospective study by Cerci et al.,[22] 9.6% (18/188) of the patients who underwent PET/CT-guided biopsies presented with FDG-avid foci and no corresponding anatomical changes on CT. The authors indicated that the importance of FDG PET/CT information in identifying biopsy sites should be emphasized. Kostakoglu et al .[23] found that a biopsy of the most accessible lesion can minimize sampling error and can reduce the likelihood of complications associated with the procedure.

PET guidance can optimize the diagnostic yield of image-guided interventions and can guide needle placement in a viable portion of the lesion. Furthermore, PET/CT-guided biopsy can visualize malignancies even before morphological changes become apparent [24]. In 2008, O’Sullivan et al .[20] first reported that in terms of musculoskeletal lesions, ^18^F-FDG PET/CT-guided biopsy can be used to localize the primary lesion, identify a site to biopsy, and evaluate metastatic lesions that require follow-up biopsies. Subsequently, Werner et al .[25] reported that in a patient who underwent extensive imaging and multiple futile biopsies (CT, MRI and bone scintigraphy), only FDG PET/CT-guided biopsy at a site of high metabolic activity yielded the final diagnosis of bone metastases. These results indicated that PET/CT-guided biopsy is a feasible, safe and efficient approach for the diagnosis of bone tumors and tumor-like lesions.

In our current study, of the 42 lesions biopsied with PET/CT guidance, only one (2.38%) was considered histologically inconclusive due to a lack of tumor cells, and the results for 41 (97.62%) were concordant with the final surgical pathology reports. The diagnostic yield and accuracy of the PET/CT group in our series are consistent with previously reported value s[15, 19]. Of the 55 lesions biopsied with CT guidance, 10 (18.18%) were considered histologically inconclusive, and 3 (5.45%) were inconsistent with the final surgical pathology reports. As a result, both the overall diagnostic yield and accuracy of PET/CT-guided biopsy were 97.62%. These results compare favorably with those of CT-guided bone biopsy (overall diagnostic yield: 81.82% and accuracy: 76.36%, respectively). We observed significant differences in terms of diagnostic performance between the two groups (P<0.05), similar to what has been suggested in the literature [11, 15, 16, 19–22]. However, our data suggest that percutaneous CT biopsy costs approximately one-third of what PET/CT biopsy costs [(13 ± 5.5 vs 4.3 ± 3.1) thousand RMB, *P* < 0.001]. The results of the current study have not been reported in similar studies [19, 21]. Our calculations were limited by the retrospective design of this study; additionally, geographical and medical differences may introduce bias in the overall cost.

In addition to confirming the high diagnostic performance of PET/CT-guided biopsy for bone tumors and tumor-like lesions, our data also revealed that of the 55 lesions biopsied with CT guidance, the bone biopsy and surgical resection results of 3 cases were considered histologically discordant. The 3 cases of discordance included one osteosarcoma (bone biopsy: fibrous histiocytoma) and two giant-cell tumors (bone biopsies: aneurysmal bone cyst and chondroblastoma, respectively). These results could be attributed to the presence of fibrous tissue, necrosis, blood or the lack of tissue. According to previously proposed studies and our experience in CT-guided biopsy, we concluded that multiple factors are involved. First, in cases of malignant bone lesions, such as osteosarcomas that contain numerous giant cells and telangiectatic osteosarcomas, the dominant tissue architecture may contain various components, such as hematomas, necrosis, and/or reactive bone formations; therefore, the pathologist requires a true-cut biopsy core, which is a core tissue sample rather than a sample from the reactive zone, to make a definitive diagnosis [16, 26]. Moreover, low-grade tumors, such as giant-cell tumors and osteoblastomas, contain heterogenic cells with nonspecific morphology and osteoclast activity and require tissue structure evaluations rather than evaluations through cellular typing. Second, hematologic malignancies, such as lymphoma and myeloma, are mainly composed of loose tissues; some of these tissues may be necrotic, such as in large-cell lymphomas, or more prone to damage during pathologic sample preparation, which interferes with immunohistochemistry-based phenotyping evaluations [1, 7, 8]. Third, some bone lesions, such as Langerhans cell histiocytosis, simple cysts and aneurysmal bone cysts, lack specific imaging features, and the similarities of their clinical characteristics with other malignancies represent diagnostic challenges for both the radiologist and pathologis t[27]. Eventually, all samples with discordant diagnoses in our series were evaluated with CT-guided biopsy, which may represent a potential limitation [13, 19]. Other limitations, such as lesion size, biopsy sites and the amount of tissue sampled, may have influenced our results. In our series, the most common histopathology results in the PET/CT group were primary bone malignancies (19/42, 45.24%) and bone metastases (17/42, 40.48%), which were more prevalent than in the CT group. However, our results were limited by patient selection; additionally, according to the specialist MDT setting, PET/CT-guided biopsy may be initially preferred for patients with suspected bone metastases or primary bone malignancies. Our experience shows that this procedure provides the most accurate and viable portion of the target lesion and enables early detection of other occult and metastatic lesions throughout the entire body.

In agreement with previously published literature, no patients in our study population showed major biopsy-related complications, but slight bleeding and pain occurred in almost all patients [6, 12, 19, 22]. Guo et al .[19] found that selecting metastatic tumors rather than primary lung tumors as the target location may minimize the occurrence of complications. The authors described that the optimal needle path is also very important, and the major blood vessels, spinal nerve trunk and vital organs should be avoided during percutaneous image-guided interventions. In both groups, our surgeons determined the optimal puncture site depending on the target location, optimal needle path and shortest skin-to-target distance. When selecting the target location of the PET/CT group, the most metabolically active part of the lesion should be selected. In contrast, choosing reactive areas with necrosis or fibrosis (which are generally less metabolically active or even inactive) usually leads to sampling errors [15, 19]. Moreover, the shortest skin-to-target distance can simplify the biopsy procedure, thereby saving time and reducing pain during the intervention [7].

Our results reflect only our practice of diagnosing bone tumors and tumor-like lesions in a population of Chinese patients, with specific indications for each technique. Limitations to this study must be acknowledged. First, the sample size of the PET/CT-guided biopsy group was limited. We attempted to retrieve multicenter data to test and verify our results. Second, only ^18^F-FDG PET/CT- and CT-guided biopsies were studied; biopsies with other modalities, such as MRI-guided biopsy, ultrasound-guided biopsy, and even open biopsy, must be considered. In a retrospective study, Kerimaa et al .[28] reported the results of MRI-guided musculoskeletal biopsy of 163 patients. Their results are comparable to those obtained with CT guidance, with 95% overall diagnostic accuracy. Interestingly, Fritz et al .[29] reported that image overlay technology can provide accurate navigation for the MRI-guided biopsy of bone lesions of the spine and pelvis in human cadavers with high technical and diagnostic yield. However, the presence of the magnetic field in MRI requires special instrumentation, which may present a major limitation to clinical treatment and add additional cost. Finally, various subtypes of bone tumors present differences in terms of PET/CT findings. In regard to these problems, more prospective randomized studies will be conducted in the future.

## Conclusion

PET/CT-guided percutaneous bone biopsy, compared with CT-guided bone biopsy, is an effective and safe alternative that yields a high diagnostic performance in the evaluation of hypermetabolic bone lesions to diagnose bone tumors and tumor-like lesions. This technique should be considered the best choice for obtaining tumor samples.

## Data Availability

The datasets used and/or analyzed during the current study are available from the corresponding author on reasonable request.

## Abbreviations

CFDA: China Food and Drug
CT: Computed tomography
FDG: ^18^F-fluorodeoxyglucose
MRI: magnetic resonance imaging
PET/CT: Positron emission tomography/computed tomography
RMB: Ren min bi
SUV: Standardized uptake value
